# Differential effects of the cell cycle inhibitor, olomoucine, on functional recovery and on responses of peri-infarct microglia and astrocytes following photothrombotic stroke in rats

**DOI:** 10.1186/s12974-021-02208-w

**Published:** 2021-07-31

**Authors:** Wai Ping Yew, Natalia D. Djukic, Jaya S. P. Jayaseelan, Richard J. Woodman, Hakan Muyderman, Neil R. Sims

**Affiliations:** 1 College of Medicine and Public Health, Flinders University, GPO Box 2100, Adelaide, SA 5001 Australia; 2 Flinders Centre for Epidemiology and Biostatistics, College of Medicine and Public Health, Flinders University, Adelaide, SA Australia

**Keywords:** Stroke, Focal ischemia, Microglia, Astrocytes, Olomoucine, Cell cycle inhibition, Functional recovery, Peri-infarct, Photothrombosis

## Abstract

**Background:**

Following stroke, changes in neuronal connectivity in tissue surrounding the infarct play an important role in both spontaneous recovery of neurological function and in treatment-induced improvements in function. Microglia and astrocytes influence this process through direct interactions with the neurons and as major determinants of the local tissue environment. Subpopulations of peri-infarct glia proliferate early after stroke providing a possible target to modify recovery. Treatment with cell cycle inhibitors can reduce infarct volume and improve functional recovery. However, it is not known whether these inhibitors can influence neurological function or alter the responses of peri-infarct glia without reducing infarction. The present study aimed to address these issues by testing the effects of the cell cycle inhibitor, olomoucine, on recovery and peri-infarct changes following photothrombotic stroke.

**Methods:**

Stroke was induced by photothrombosis in the forelimb sensorimotor cortex in Sprague-Dawley rats. Olomoucine was administered at 1 h and 24 h after stroke induction. Forelimb function was monitored up to 29 days. The effects of olomoucine on glial cell responses in peri-infarct tissue were evaluated using immunohistochemistry and Western blotting.

**Results:**

Olomoucine treatment did not significantly affect maximal infarct volume. Recovery of the affected forelimb on a placing test was impaired in olomoucine-treated rats, whereas recovery in a skilled reaching test was substantially improved. Olomoucine treatment produced small changes in aspects of Iba1 immunolabelling and in the number of CD68-positive cells in cerebral cortex but did not selectively modify responses in peri-infarct tissue. The content of the astrocytic protein, vimentin, was reduced by 30% in the region of the lesion in olomoucine-treated rats.

**Conclusions:**

Olomoucine treatment modified functional recovery in the absence of significant changes in infarct volume. The effects on recovery were markedly test dependent, adding to evidence that skilled tasks requiring specific training and general measures of motor function can be differentially modified by some interventions. The altered recovery was not associated with specific changes in key responses of peri-infarct microglia, even though these cells were considered a likely target for early olomoucine treatment. Changes detected in peri-infarct reactive astrogliosis could contribute to the altered patterns of functional recovery.

**Supplementary Information:**

The online version contains supplementary material available at 10.1186/s12974-021-02208-w.

## Background

Stroke is a leading cause of adult disability [1]. Ischemic stroke, which mostly results from occlusion of a major cerebral artery, accounts for more than 70% of cases worldwide [1]. Arterial occlusion in the brain leads to local severe reductions in blood flow because of the limited overlap of perfusion territories of the vessels. If the occlusion is not rapidly reversed, an infarct develops due to the death of essentially all cells in the affected tissue [2, 3]. The location of the infarct is the primary determinant of the symptoms that are seen.

Many of those affected by stroke show improvements in neurological function within the first few weeks to months after disease onset [4]. Changes in the connectivity and function of neurons in the “peri-infarct tissue” which surrounds the infarct, as well as at more distant sites, are important contributors to these improvements [5–8]. Treatments promoting these adaptive responses have the potential to provide effective therapies that could be initiated well after stroke onset [4, 7, 8].

The focal ischemic insult and infarct formation trigger a complex chain of cellular responses in surrounding tissue. Microglia respond rapidly with prominent morphological changes that include retraction of the cellular processes and enlargement of the soma [9–11]. Microglial numbers around the injured tissue increase [11, 12] due to local proliferation as well as migration from surrounding regions [13, 14]. The microglia release cytokines and chemokines that interact with neighbouring cells and exhibit other changes in function that are associated with progressive modifications in the pattern of gene expression [13, 14].

Responses of astrocytes in peri-infarct tissue are initiated within the first day or two of stroke onset, at least in part as a response to signals from the microglia [15–18]. The astrocytes greatly increase expression of the cytoskeletal proteins, GFAP and vimentin, which are hallmarks of reactive astrogliosis in injured brain tissue [17, 18]. Astrocytes immediately adjacent to the infarct undergo morphological and functional changes that initiate formation of an astroglial scar around the infarct. A subpopulation of these astrocytes proliferates in the first few days after stroke [12, 19, 20], a process that is apparently essential for the development of the scar [15, 21].

Astrocytes in tissue extending well beyond the developing glial scar also exhibit features of reactive astrogliosis that persist for many weeks [16–18]. These cells generally do not proliferate [12, 19, 22]. Changes in properties of these astrocytes can more directly influence the adaptive responses of surviving neurons in the peri-infarct tissue.

The proliferation of microglia and astrocytes within the first hours to days after stroke are key elements of tissue changes that influence adaptive responses in peri-infarct tissue and functional recovery. The manipulation of these glial cell changes has the potential to improve outcomes. Treatment with the cell cycle inhibitors, olomoucine and roscovitine, initiated shortly before or immediately after temporary middle cerebral artery occlusion can result in smaller infarct volumes [23–26], reductions in aspects of peri-infarct reactive astrogliosis and microglial reactivity [23, 27, 28] and improved recovery of neurological function [23, 25, 26]. Decreases in infarct volume often lead directly to smaller peri-infarct glial cell responses and improvements in functional recovery. Thus, these studies mostly do not address the question of whether treatment with cell cycle inhibitors can modify glial cell responses or influence functional recovery independently of effects on infarct size. In one investigation, treatment with olomoucine, a cell cycle inhibitor that blocks multiple cyclin-dependent kinases [29, 30], did decrease GFAP expression and astrocyte proliferation without significantly affecting infarct size following short-term middle cerebral artery occlusion [28]. This finding suggests that olomoucine can have a more direct effect on responses of peri-infarct reactive astrogliosis. However, possible changes in microglial responses and the consequences for functional recovery were not investigated.

In the present study, we tested whether olomoucine can affect key responses of peri-infarct microglia and astrocytes and improve recovery in a photothrombotic model of stroke [31] in which the infarct was localized to the sensorimotor cortex of rats. A small infarct was generated, modelling the situation in humans that is associated with better functional recovery [5, 32]. Photothrombotic stroke has often been used to characterize neuronal changes contributing to recovery of neurological function following stroke [5, 33]. Furthermore, the infarct develops rapidly and reaches maximal volume within the first 24 h under the conditions used in the present study [11]. Thus, the potential for treatment-induced changes in this tissue damage is substantially reduced compared with models involving occlusion of a major artery.

The effect of olomoucine on forelimb function was assessed using a placing test and two components of a single-pellet skilled reaching test. The forelimb placing test examines a motor response to stimulation of the vibrissae that provides a sensitive measure of general limb function following photothrombotic stroke [11]. The skilled reaching test involves a more complex learned response in the rats [34].

Two aspects of Iba1 immunolabelling were used as the primary indicator of the responses of microglia in peri-infarct tissue at 3 days after stroke, a time when changes in these properties are near maximal [11]. The use of immunohistochemistry ensured that cells in the peri-infarct tissue could be evaluated without contamination from microglia in the infarct. Tissue macrophages derived from the circulation also express Iba1. However, microglia substantially outnumber the macrophages in peri-infarct tissue at 3 days after photothrombotic stroke (see “Discussion” section) and are the primary contributors to Iba1 immunolabelling at this time.

Increases in expression of GFAP and vimentin, indicative of astrogliosis, develop more slowly and continue for longer than changes in Iba1 immunolabelling in the peri-infarct tissue [11]. Possible effects on the content of these proteins were assessed as the primary indicators of astrogliosis at 7 days when the responses approach maximal. Olomoucine treatment was previously shown to result in decreases in GFAP content at 7 days but not at earlier times following short-term middle cerebral artery occlusion [28]. In contrast to Iba1-positive cells, astrocytes and their proteins are largely absent from the infarct. Thus, Western blot analysis of the infarct and surrounding tissue provides a convenient means of measuring the marked increases in expression of the astrocytic proteins in the peri-infarct tissue [11].

## Methods

### Experimental design

Male Sprague-Dawley rats were obtained from Laboratory Animal Services (University of Adelaide, Adelaide, Australia). The animals were kept in a temperature-controlled and humidity-controlled room with a 12 h/12 h light/dark cycle.

**Fig. 1:**
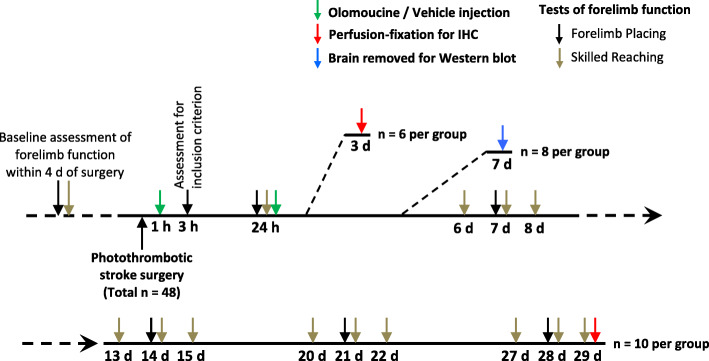
Summary of experimental design. Olomoucine was injected at 1 h and 24 h after stroke induction. The effects on forelimb function were assessed up to 29 days and different aspects of cellular responses in brain tissue were assessed at 3, 7, and 29 days after the stroke. The numbers of rats used in different parts of the study are summarized in the figure. For the brains fixed at 29 days, coronal sections prepared from 6 rats per treatment group were subsequently analyzed following immunolabelling to detect GFAP and collagen IV

A total of 50 rats were used in these investigations. One of these rats died during induction of anesthesia and a second at the conclusion of the period of light exposure. The other 48 rats successfully completed the process for stroke induction and met an inclusion criterion for the study based on performance in a forelimb placing test (see below). Of these rats, 20 were used for assessment of forepaw function up to 29 days after stroke; 12 for studies of Iba1, CD68 and Ki67 immunolabelling and infarct volume at 3 days; and 16 for Western blot analysis at 7 days. Sections of brains removed at 29 days from 12 rats were used for studies of GFAP and collagen IV immunolabelling. Figure 1 summarizes the experimental design and numbers of rats used in each part of the study.

Rats weighed between 270 and 340 g at the time of stroke induction. There was no significant difference between the two treatment groups in the weights of the rats for any of the investigations: forepaw function – olomoucine-treated: 293 ± 9 g (mean ± S.D.), vehicle-treated: 300 ± 16 g; immunohistochemistry and infarct volume – olomoucine-treated: 301 ± 14 g, vehicle-treated: 290 ± 9 g; Western blot analysis: – olomoucine-treated: 288 ± 7 g, vehicle-treated: 286 ± 11 g.

Rats were randomly pre-assigned to treatment with either olomoucine or vehicle. All assessments of forepaw function as well as the imaging and analysis of brain sections and Western blots were performed by investigators who were blinded to the treatment.

### Induction of focal ischemia by photothrombosis

An infarct was produced in the forelimb motor cortex using minor modifications [11] of previously described procedures [31, 35]. Rats were initially anesthetized with an intraperitoneal injection of 100 mg/kg ketamine and 10 mg/kg xylazine. Anesthesia was maintained as required by additional injections of ketamine via a femoral vein cannula. Each rat was placed in a stereotaxic frame and the surface of the skull exposed. A brass shim stencil with a rectangular window (3 × 5 mm) was centred at 1.5 mm anterior to bregma and 2.5 mm lateral to the midline on the side contralateral to the limb that was used preferentially by the rat in a single pellet retrieval task (as discussed below). The stencil ensured that tissue in the region of the forelimb motor cortex was the target for light exposure.

A 150 W light source (Intralux 5000; Volpi AG, Schlieren, Switzerland) equipped with a green filter (532.5–587.5 nm pass band) and attached fibre-optic cable (6 mm diameter) was used to illuminate the skull. Before each use, the intensity of the light source was measured at the point of emission from the fibre-optic cable and ranged between 155,000 and 172,000 lx. There was no significant difference in the mean light intensity between the two treatment groups for the three main elements of the investigations. The fibre-optic cable was positioned directly over the window of the stencil and as close as possible to the skull without contacting it. Rose bengal (10 mg/ml, 13 mg/kg) was injected at 200 μl/min via the femoral vein cannula. The light source was then immediately activated and remained illuminated for 15 min.

### Olomoucine treatment

Rats were treated with olomoucine using a minor modification of the protocol of Wang et al. [28]. A stock solution of 50 mg/ml olomoucine was prepared in DMSO and stored in aliquots at − 20 °C. Immediately before injection, the stock was diluted with water to 1 mg/ml. Rats were injected intraperitoneally with either olomoucine (5 mg/kg) or vehicle (DMSO in water) at 1 h and 24 h after the conclusion of the light exposure to induce photothrombosis.

### Assessment of forelimb function

The forelimb function of rats was assessed up to 29 days after stroke induction using a forelimb placing test and two components of a single pellet skilled reaching test.

#### Forelimb placing test

A minor modification [11] of the forelimb placing response as described by Schallert and Woodlee [34] was used as a criterion for inclusion in the study and as a measure of recovery of forelimb motor function after stroke. No specific training was needed for this task. However, rats were familiarized with the procedure for at least 10 min per day on three separate days leading up to stroke induction. In these sessions, rats were placed on the testing platform for a few minutes and were given approximately 5 trials of each variant of the forelimb placing test.

Three variants of the test were used [11, 34]. The rat was first moved towards the platform so that both sets of vibrissae were stimulated. If a rat placed the forelimb being tested on the platform, the trial was scored as a success. Stimulation of the vibrissae ipsilateral and then contralateral to the limb being assessed was used in subsequent tests. For each forelimb, a score out of 30 was determined from the sum of successes with 10 trials of each of the three variants of the test.

A baseline value for this study was obtained within four days of stroke induction. Rats were tested again at 3 h and then at 1, 7, 14, 21 and 28 days after stroke. A score of 5 or less for the limb contralateral to the infarct at 3 h after stroke was used as an inclusion criterion for the study. This criterion was met by all 48 rats that survived the procedure for stroke induction.

#### Single pellet skilled reaching test

Rats were trained to retrieve a pellet through a narrow slit in a single pellet reaching box and tested at baseline and up to 29 days after stroke for total success and first attempt success in retrievals using the forelimb contralateral to the infarct as described previously [35, 36]. To facilitate training, rats were food restricted so that their body weight gradually reached 90 to 95% of that expected if they had free access to food. Rats were trained in daily 10-min sessions over 2 to 3 weeks up to the day prior to stroke induction. Initial training allowed the paw preference for this task to be identified. Baseline scores are the average from two tests performed on separate days within 4 days of stroke induction, including one test on the day preceding the induction. The average baseline success for pellet retrieval exceeded 50% for all rats.

In a previous study, recovery on this task was highly variable when rats were only tested at weekly intervals after stroke [11]. To improve reproducibility of the findings and potentially promote recovery in this study, rats were tested on day 1 and then in blocks of three consecutive days centred on days 7, 14, 21 and 28. The values reported for days 7 to 28 are the average of the scores on the three consecutive days. On day 1, rats showed reduced interest in pellet retrieval. Testing on this day was terminated if 20 trials were not completed within 15 min.

### Brain fixation and assessment of infarct volume

Brain tissue from 12 rats was fixed for immunohistochemistry and analysis of infarct volume at 3 days after stroke. The brains of the 20 rats that were used to test recovery of forelimb function were fixed for additional immunohistochemical analysis on day 29. Rats were anesthetized with 100 mg/kg ketamine and 10 mg/kg xylazine injected intraperitoneally. The brains were perfusion fixed and prepared for sectioning as described previously [11]. Coronal sections (20 μm) were prepared using a cryo-microtome (Leica Systems, NSW, Australia).

Infarct volume was determined at 3 days after stroke induction using cresyl-violet stained sections at 0.5 mm intervals through the infarct, as described previously [11].

### Antibodies

Antibodies for immunohistochemistry to detect Iba1 (goat polyclonal; catalogue number ab5076), Ki67 (rabbit monoclonal; clone SP6; catalogue number ab16667), CD68 (rabbit polyclonal; catalogue number ab125212) and collagen IV (rabbit polyclonal; catalogue number ab6586) were from Abcam (Cambridge, UK). The antibody for detecting GFAP (rabbit polyclonal; catalogue number G4546) was from Sigma-Aldrich (St Louis, Mo) and for NeuN (mouse monoclonal; clone A60; catalogue number MAB377) was from Millipore-Chemicon (Merck, Darmstadt, Germany). Alexa-fluor-conjugated secondary antibodies were from Thermo Fisher Scientific (Waltham, MA, USA).

For Western blot analysis, antibodies to detect GFAP (mouse monoclonal; clone G-A-5; catalogue number G3893) were from Sigma-Aldrich, those for vimentin (rabbit monoclonal; clone EPR3776; catalogue number ab92547) were from Abcam and for neurocan (mouse monoclonal; clone 650.24; catalogue number MAB5212) were from Millipore-Chemicon. A peroxidase donkey anti-rabbit IgG (catalogue number: 711-035-712) from Jackson ImmunoResearch was used as secondary antibody.

### Immunohistochemistry

Two brain sections separated by at least 500 μm were selected from each sample for immunolabelling with each marker. Brain sections from 3 days after stroke were used to assess possible changes in the properties of microglia and tissue macrophages by immunolabelling for Iba1 together with either CD68 or Ki67. Brain sections from 29 days after stroke were used in the investigation of longer-term changes in astrogliosis and angiogenesis using antibodies against GFAP and collagen IV respectively. All sections were also co-labelled with the NeuN antibody to visualize the infarct and assist in identifying the peri-infarct region for analysis (see “Image acquisition and analysis” section).

The protocols for immunohistochemistry are essentially as described in detail in previous studies [11, 37]. Free-floating brain sections were blocked and permeabilized in phosphate-buffered saline (PBS) containing 0.3% Triton X-100 and 5% donkey serum (blocking buffer) for 2 h at room temperature, then incubated overnight at 4 °C in primary antibodies diluted in blocking buffer (Iba1, 1/800; CD68 and collagen IV, 1/500; Ki67, GFAP and NeuN, 1/400). The sections were then washed in PBS and incubated in secondary antibodies diluted (1/2000) in PBS for 2 h at room temperature on an orbital shaker. Finally, the sections were washed in PBS and mounted on glass slides in Prolong Gold antifade mountant (Thermo Fisher Scientific). Sections to be immunolabelled for collagen IV were incubated prior to processing for immunohistochemistry in 10 mM citrate buffer (pH 8.5) for 10 min at 85 °C to promote antigen retrieval.

### Image acquisition and analysis

The methods for image acquisition and analysis used in the present study were largely as described in detail previously [11, 37]. Minor modifications were made in the image processing and analysis parameters to account for differences in image resolution and quality due to the different microscopy systems employed between the studies.

Whole immunolabelled sections, other than those immunolabelled for collagen IV, were batch-scanned using the Pannoramic 250 Flash II slide scanner (3DHistech; Budapest, Hungary). Images from the peri-infarct and equivalent contralateral regions were then extracted using the CaseViewer software (ver 2.1; 3DHistech) into TIFF files for analysis in ImageJ (ver 1.51j8) [38].

For sections immunolabelled for collagen IV, images were acquired using an Olympus IX71 inverted microscope with the 10× objective lens. Two adjacent images were captured and stitched together in ImageJ for each hemisphere to create a field of view covering the entire infarct and the equivalent contralateral region.

Responses of microglia and macrophages within the peri-infarct tissue were assessed using the parameters of circularity and area fraction of particles in Iba1-immunolabelled sections. Two rectangular regions of interest (ROIs) were analyzed. These encompassed the entire cortical layers extending 500 μm from the lateral edge of the infarct beginning where NeuN immunoreactivity was preserved). Two corresponding ROIs in the contralateral hemisphere were also analyzed (see Fig. 3A). Images were processed in ImageJ using the “subtract background” function with “rolling ball radius” set at 50 pixels and the “sliding paraboloid"” option selected. The two ROIs were then thresholded using the “Huang” method and the circularity and area fraction of Iba1-positive particles were determined using “Analyze particles” with the particle size set at 2000-infinity pixels (equivalent to particles with diameters of approximately 8 μm and greater) and circularity at 0.00–1.00.

Counts for CD68-positive and Ki67-positive particles were assessed using ROIs defined using the same approach as for Iba1 and background subtraction was performed using the same parameters. The images were thresholded using the “Otsu” method and the particles analyzed with particle size set at 120-infinity pixels (diameter 2 μm or greater) and circularity at 0.00–1.00.

The results for CD68 and Ki67 from these analyses for each brain sample are the average of two sections. Iba1 was co-labelled with each of these two markers resulting in four sections analyzed and averaged for each brain sample for the circularity and area fraction analyses.

GFAP and collagen IV immunolabelling were analyzed using methods described previously [37]. The area fraction of GFAP in the peri-infarct tissue was assessed in four adjoining 250-μm-wide rectangular ROIs on the lateral edge of the infarct as well as in corresponding ROIs in the contralateral hemisphere (see Fig. 5A). As reported previously [37], the changes in collagen IV immunolabelling was limited to regions very close to the edge of the infarct and therefore analysis of this marker was only performed in two adjacent rectangular ROIs extending 500 μm perpendicular to the lateral edge of the infarct.

For analysis of GFAP immunolabelling, the raw unprocessed images were analyzed. The ROIs within the contralateral region were thresholded using the “Triangle” method in ImageJ and the same thresholding level was applied to the ROIs within the peri-infarct region. The area fraction of GFAP immunolabelling was then determined using the “Measure” function.

For collagen IV labelling, background noise was first reduced by applying the “subtract background” function with “rolling ball radius” set at 5 pixels and “sliding paraboloid” option selected, after which the “Despeckle” function was applied. The ROIs within the peri-infarct tissue and equivalent contralateral regions were thresholded using the “Li” method and the area fraction of collagen IV immunolabelling within each ROI was determined using the “Analyze Particle” function with the particle size set to a lower limit of 20 pixels (to exclude debris).

### Western blot analysis

Rats were decapitated under anesthesia induced by intraperitoneal injection of ketamine (100 mg/kg) and xylazine (10 mg/kg) at 7 days after stroke induction. For each rat, the brain was rapidly removed from the skull and the infarct was identified from the paleness of the tissue on the surface of the cortex. Cerebral cortex containing the infarct plus approximately 2 mm of surrounding tissue was dissected out for analysis.

The tissue samples were homogenized in a buffer containing 10 mM Tris, 1 mM EDTA, 0.32 M sucrose, pH 7.4 and a protease inhibitor cocktail (Sigma-Aldrich) using a Retsch TissueLyser (Qiagen, Chadstone, Vic, Australia) at a frequency of 30 Hz for 2 min. Western blot analysis for the vimentin, GFAP and neurocan were as described previously [11]. Total protein in the homogenates was determined using a Bradford protein assay kit (Bio-Rad Laboratories, Gladesville, NSW, Australia). Samples (20 μl) containing 20 μg protein were loaded for gel electrophoresis on 4–20% Criterion™ TGX Stain-Free™ Protein gels, (#5678094; Bio-Rad Laboratories).

Samples for Western blot analysis were initially prepared from 6 rats in each of the two treatment groups. Although all samples were handled identically, in initial investigations to detect vimentin and GFAP, two samples from olomoucine-treated rats showed a substantially reduced content of these proteins and additional immunoreactive bands at lower molecular weights indicating protein degradation (as seen in the GFAP blot in Additional file 1: Fig. S1). Two further samples from each treatment were included in subsequent analyses. One of these from a vehicle-treated rat also showed degradation. The three degraded samples were not included in the subsequent analysis. Thus, results are presented for six rats treated with olomoucine and seven with vehicle. Results for vimentin and neurocan were based on analysis of all samples run in single blots. Two blots were used for GFAP analysis with band intensities normalized based on the average values for the five samples that were included in both blots.

Stain-free images following protein transfer to the polyvinylidene fluoride membrane were obtained using a Gel Doc™ EZ Imager (Bio-Rad Laboratories) and the intensity of protein labelling in a full-length transect of each lane was measured as described by Colella et al. [39]. Chemiluminescence from the blots following antibody treatment was captured digitally on a Gel Doc™ EZ Imager and the band intensities analyzed using the Carestream Molecular Imaging Software (Version 5.0; Carestream Health, NY, USA). The intensity of vimentin or GFAP bands were expressed relative to total protein in each lane as determined from the stain free images. For neurocan, the intensity of the band for full-length protein, which increases markedly in response to tissue damage [40–42], was determined as a ratio to a fragment of this protein with a molecular weight of approximately 150 kDa that is expressed in normal brain and is largely preserved during the response to damage [40–42].

### Statistical analysis

Analysis was performed using SPSS (version 23.0; IBM Corp., Armonk, NY, USA) and Stata (version 15.1; StataCorp College Station TX, USA).

Results from the tests of forelimb function are presented as box plots. The effect of olomoucine treatment on recovery of forelimb function was analyzed using linear mixed effects models. The models included both fixed effect and random effect terms. The random effects component consisted of a random intercept for each rat, thereby allowing variability around the overall fixed effect for each of the individual rats and accounting for the correlation across time for each rat. The fixed effects terms consisted of the day, which was treated as categorical variable and a day X treatment interaction term, in order to test whether the recovery curves differed between treatments across time. When testing for the significance of the interaction, we first tested for global significance of the day X treatment interaction terms, and, when significant, then assessed at which of the time points the difference in treatment effects occurred using the separate day X treatment interaction terms from the model. For analysis of the forelimb placing test, data for all assessments from 3 h to 28 days after stroke were used. For the skilled reaching test, initial post-stroke performance was approximated by assigning a constant value of 3 for total success and 1 for first attempt success, based on values obtained on day 1 (see “Results” section).

The results for infarct volume, parameters derived from analysis of the immunohistochemistry and band intensity of the Western blots are shown as mean ± standard deviation. The effects of olomoucine treatment on infarct volumes and band intensities from the Western blots were assessed using Student’s *t* tests. For the data obtained from analysis of images of immunolabelled brain sections, the effects of olomoucine treatment and distance from the infarct (or linear distance from an equivalent starting point within the contralateral region) were analyzed using two-way analysis of variance for each brain hemisphere. Results for immunolabelling with CD68 and Ki67 were analyzed after log transformation as the untransformed data showed significant differences in variance across the groups.

A two-sided type 1 error rate of alpha = 0.05 was used for all hypothesis testing.

## Results

### Infarct volume

There was no significant difference in infarct volume (*p* = 0.604; Student’s *t* test) between the brains of rats treated with olomoucine (17.8 ± 2.3 mm^3^) and those treated with vehicle (16.8 ± 4.0 mm^3^) when assessed at 3 days after stroke.

### Recovery of forepaw function

**Fig. 2:**
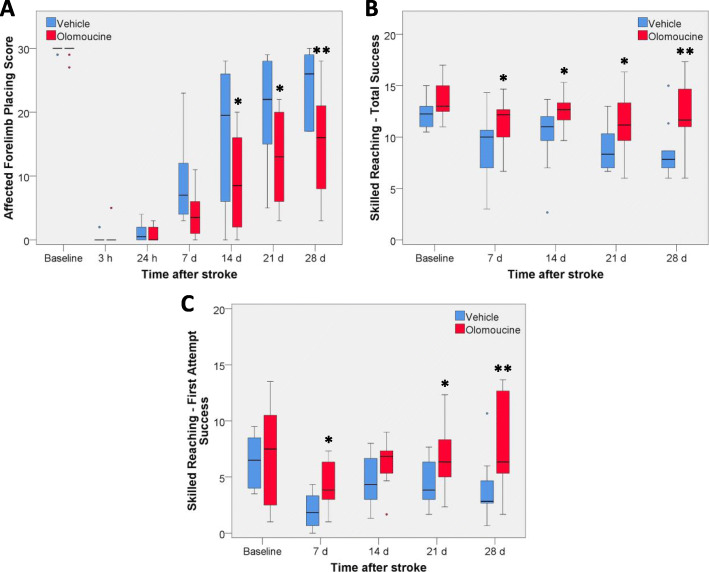
Effect of olomoucine on forelimb function following photothrombotic stroke. Box plots of dysfunction and recovery up to 28 days after photothrombotic stroke in **A** forelimb placing test, **B** single pellet skilled reaching test: total success from 20 trials, and **C** single pellet skilled reaching test: first attempt success from 20 trials. Values are for 10 rats per group. Outliers are shown as circles. For each of the tests, there was no statistically significant difference between the two treatment groups in the baseline values obtained prior to stroke induction (Mann-Whitney test). There was a difference in recovery between the olomoucine-treated and vehicle-treated rats identified from a statistically significant overall day X treatment interaction for the forelimb placing test (*p* < 0.02) and for both total success (*p* < 0.05) and first attempt success (*p* < 0.01) in the skilled reaching test (Linear mixed effects models). *p < 0.05, **p < 0.01 identifies individual days in which there was a significant difference between the two treatment groups

A forelimb placing test involving a motor response of the forelimb to vibrissae stimulation was used as an inclusion criterion at 3 h after induction of the stroke and to assess recovery over the subsequent 4 weeks. Prior to surgery, all rats scored at or close to 30 (i.e. 30 successful placements from 30 trials on this test; Fig. 2A). Within 3 h of surgery, this response was completely abolished (a score of 0) for the forelimb contralateral to the infarct in 17 of the 20 rats and gave a score of 5 or less in the other three rats. At 24 h, the performance on this test for all rats from both treatment groups remained almost completely impaired, with scores between 0 and 4. Median scores in the two groups improved over the next 4 weeks although most rats did not fully regain their pre-stroke scores (Fig. 2A). Performance of the forelimb ipsilateral to the infarct was not significantly affected by the stroke, with median scores for this forelimb between 28.5 and 30 on each day of testing.

Overall recovery on this task for the affected forelimb was significantly worse for the rats treated with olomoucine compared with the vehicle-treated group (*p* < 0.02 for the overall day X treatment interaction term). Performance between the two groups was significantly different on days 14, 21 and 28 (Fig. 2A).

The effect of olomoucine treatment on recovery of forepaw function was also assessed using a single pellet skilled-reaching test. Both the total number of successes out of 20 (irrespective of the number of attempts required for each pellet) and the number of successes at the first attempt out of 20 were evaluated (Fig. 2B, C). Performance on both aspects of this task was initially greatly affected by the stroke. When tested on day 1, only one rat in each group completed 20 trials within 15 min. The median value for the olomoucine-treated rats was 9 total attempts with 0 total successes or first-attempt successes. For the vehicle-treated group, the median values were 12 total attempts with 1.5 total successes and 0.5 first-attempt successes. Because of the failure of most rats to complete 20 trials, the results for day 1 have not been included in Fig. 2B, C*.*

The total number of successes showed more rapid recovery than the number of first attempt successes during the first 7 days. In contrast to the findings with the forelimb placing test, olomoucine treatment led to improved recovery on both the total success (*p* < 0.05 for overall day X treatment interaction) and first-attempt success (*p* < 0.01 for overall day X treatment interaction) in the skilled reaching test (Fig. 2B, C). Statistically significant differences between the treatment groups were seen at days 7, 21 and 28 for both aspects of this test and also at day 14 for total success.

### Cellular changes in peri-infarct tissue

**Fig. 3:**
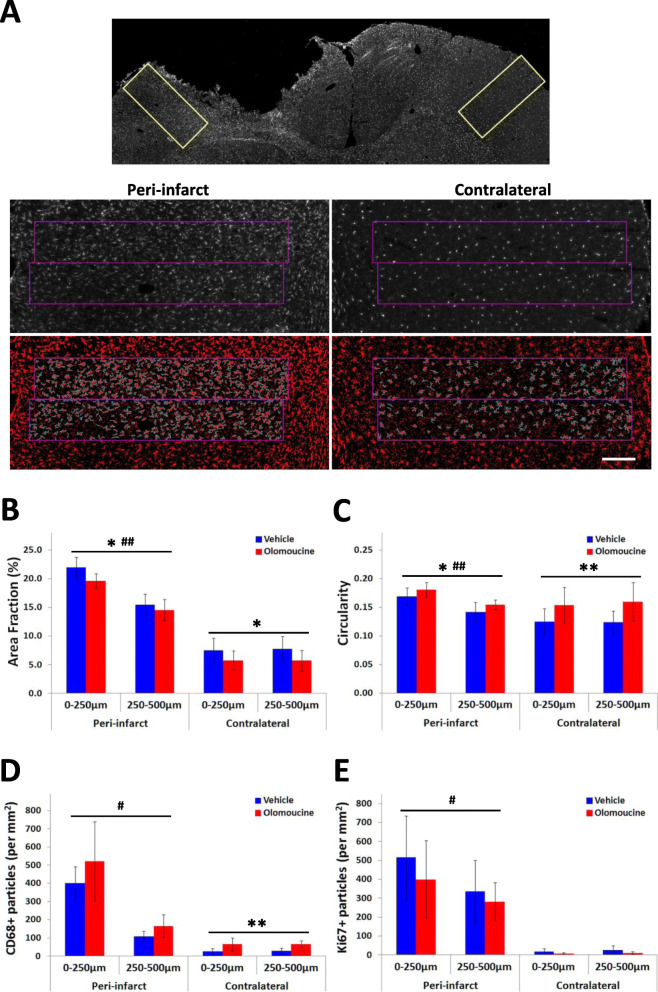
Effect of olomoucine treatment on immunolabelling for Iba1, CD68, and Ki67 at 3 days after stroke. **A** The top panel shows Iba1 immunolabelling in a coronal section including the infarct and surrounding cortex and equivalent tissue in the contralateral hemisphere. The rectangles indicate the location of the images in the lower panels, which have been processed for analysis of the area fraction of Iba1 immunolabelling and circularity of Iba1-positive cells. The scale bar represents 200 μm. Representative unprocessed images are shown in Additional file1; Fig. S2. **B**, **C** The effect of treatment on the area fraction of Iba1 immunolabelling (**B**) and circularity of Iba1-immunolabelled cells (**C**). For both measures, there was a significant effect of treatment (*p* < 0.05) and distance from the lesion (*p* < 0.01) in the peri-infarct tissue with no significant interaction. There was also a significant effect of treatment but not location in the contralateral hemisphere. **D**. The effect of treatment on CD68-positive cells. In the peri-infarct tissue, there was a significant effect of distance (*p* < 0.05) but not treatment (*p* = 0.065), whereas the contralateral cortex showed a significant effect of treatment (*p* < 0.01) but not tissue location. **E** The effect of treatment on Ki67-positive cells. This marker differed significantly with distance from the lesion in peri-infarct tissue (*p* < 0.05) Olomoucine did not significantly affect the numbers of Ki67-positive cells in either hemisphere. Results in **B** to **E** are presented as mean ± SD (*n* = 6 rats per treatment group) with data for each hemisphere analyzed using two-way analysis of variance. *p < 0.05; **p < 0.01 for the effect of olomoucine treatment. #*p* < 0.05; ##*p* < 0.01 for the effect of distance from the infarct or of location in the contralateral cortex

Key aspects of the response of microglia and blood-derived macrophages were assessed from the pattern of Iba1 expression at 3 days when changes are near maximal following photothrombotic stroke [11]. Consistent with previous findings [11], the area fraction of Iba1 immunolabelling was several-fold higher in peri-infarct tissue compared with equivalent tissue in the contralateral cerebral cortex (Fig. 3A, B). This increase was most obvious within the first 250 μm from the infarct but also extended into surrounding tissue. Treatment with olomoucine significantly reduced the area fraction of Iba1 immunolabelling in peri-infarct tissue (Fig. 3B). However, this change was small relative to the total change in area fraction. Furthermore, a similar reduction was seen in tissue from the contralateral hemisphere. Thus, the increase attributable to the development of the infarct was similar in the rats treated with olomoucine and those treated with vehicle.

The circularity of Iba1 immunolabelled cells provides a more direct measure of the changes in microglial morphology that occur during activation of these cells. Values for this parameter increase in peri-infarct tissue within the first day after stroke due to retraction of processes and increases in the volume of the cell body [11]. Increases in the circularity of Iba1-immunolabelled cells in peri-infarct tissue compared with the contralateral cortex were again most prominent within 250 μm of the infarct (Fig. 3C). Treatment with olomoucine significantly increased the circularity of Iba1 immunolabelled cells but again a similar change was seen in both peri-infarct tissue and equivalent tissue in the contralateral hemisphere (Fig. 3C).

The effects of olmoucine on the number of cells in peri-infarct tissue expressing CD68 was also investigated. CD68 is a protein produced by subpopulations of Iba1-positive cells within the first few days of stroke induction. Reductions in these cells have been seen previously following various treatments that led to improved functional recovery [11, 43–45]. The increases in CD68-positive cells were more restricted in distribution than those for Iba1 immunolabelling, resulting in relatively larger increases in the tissue immediately surrounding the infarct (Fig. 3D). Within the initial 250 μm of this tissue, 84 ± 7% of the CD68-positive cells were also Iba1-positive. Very low numbers of CD68-positive cells were detected in the equivalent tissue from the contralateral hemisphere but most (71 ± 17%) were again Iba1-positive. Olomoucine treatment did not result in statistically significant differences in the number of CD68-positive cells in peri-infarct tissue (Fig. 3D), although there was a trend towards an increase (*p* = 0.065). In equivalent tissue from the contralateral hemisphere, a statistically significant increase (*p* < 0.01) in the numbers of these cells was seen in the olomoucine-treated rats.

Immunolabelling for the cell-cycle protein, Ki67, was also measured at 3 days to assess possible downstream changes in cell proliferation resulting from the olomoucine treatment. There was substantial variability in Ki67 immunolabelling between rats but cells containing Ki67 were again most prominent in tissue adjacent to the infarct (Fig. 3E). Very few immunolabelled cells were seen in the contralateral hemisphere (Fig. 3E). There was no statistically significant difference between the olomoucine-treated and vehicle-treated groups. Cells labelled with Iba1 were a substantial component of the Ki67-positive cells, particularly close to the infarct. There was again no significant effect of olomoucine treatment on the proportion of these cells relative to total Ki67-positive cells (olomoucine-treated: 35 ± 10% at 0 to 250 μm, 20 ± 13% at 250 to 500 μm; vehicle-treated: 41 ± 5% at 0 to 250 μm, 24 ± 8% at 250 to 500 μm) or on the number of these double-labelled cells (olomoucine-treated: 158 ± 126 particles per mm^2^ at 0 to 250 μm, 69 ± 71 particles per mm^2^ at 250 to 500 μm; vehicle-treated, 217 ± 110 particles per mm^2^ at 0 to 250 μm, 92 ± 71 particles per mm^2^ at 250 to 500 μm).

**Fig. 4:**
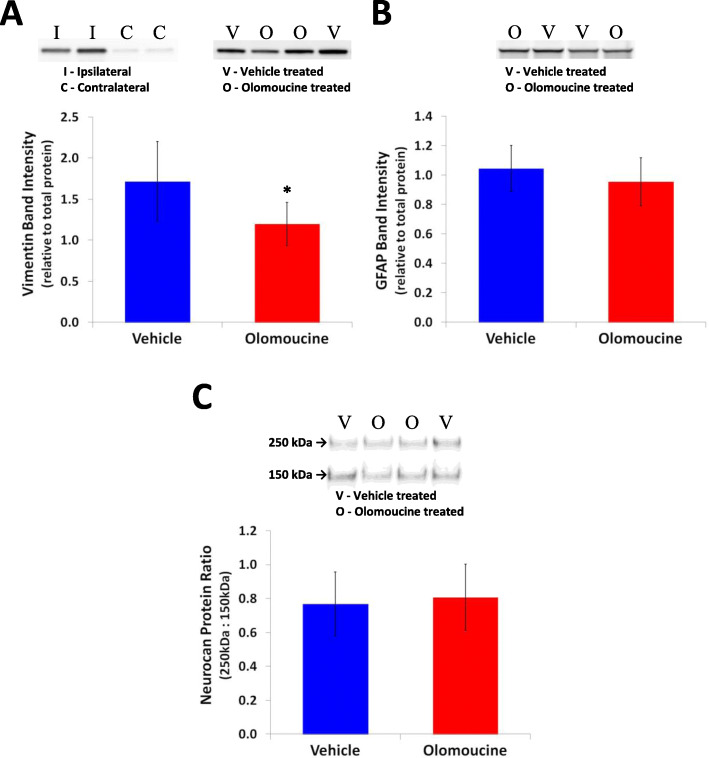
Effects of olomoucine treatment on markers of reactive astrogliosis at 7 days after stroke. **A** Vimentin: upper left image shows representative lanes from a blot comparing samples containing the infarct and peri-infarct tissue (I) and equivalent tissue from the contralateral hemisphere (C) from two rats; the upper right image shows representative lanes from a blot of infarct plus peri-infarct tissue from two rats treated with olomoucine (O) and two treated with vehicle (V). The bar graph shows the effect of olomoucine treatment on intensity of the vimentin band expressed relative to total protein in that sample. *p < 0.05 (Student’s *t* test). **B** GFAP: representative lanes from a blot of infarct plus peri-infarct tissue from two rats treated with olomoucine (O) and two treated with vehicle (V). The bar graph shows the effect of olomoucine treatment on the pooled data for intensity of the GFAP band expressed relative to total protein in that sample. The treatment did not produce a statistically significant difference in GFAP content (Student’s *t* test). **C** Neurocan: representative lanes from a blot of infarct plus peri-infarct tissue from two rats treated with olomoucine (O) or two treated with vehicle (V). The bar graph shows the effect of olomoucine treatment on the intensity of the bands for full-length neurocan (approx. 250 kDa) expressed relative to that of a proteolytic fragment of this protein (approx. 150 kDa). The treatment did not produce a statistically significant difference in this ratio (Student’s *t* test). Group results in panels **A** to **C** are shown as mean ± SD (*n* = 6 to 7 per group). Additional file 1: Fig. S1 and S3 shows the full blots including the representative lanes for treatment effects in panels **A** to **C**

The content of the intermediate filament protein, vimentin, was measured using Western blots at 7 days in samples of cerebral cortex that included the infarct and adjacent peri-infarct tissue. Vimentin is not normally expressed in astrocytes in mature brain but expression increases greatly in peri-infarct astrocytes during the first week after stroke [11, 46, 47]. Thus, changes in this protein provide a sensitive indicator of reactive astrogliosis. Preliminary analysis confirmed that samples containing the infarct and the immediately surrounding tissue contain much more vimentin than samples from the equivalent region of the contralateral cortex (Fig. 4A). Vimentin content was significantly decreased (to 70 ± 15%; *p* < 0.05) in tissue from olomoucine-treated rats compared with that from vehicle-treated rats (Fig. 4A; Additional file 1: Fig. S3). The content of GFAP, a second intermediate filament protein that also increases in reactive astrocytes in peri-infarct tissue [46, 48, 49], was not significantly different between the two groups (Fig. 4B; Additional file 1: Fig. S1). Astrocytes are also an important contributor to increases in production of full-length neurocan following stroke and other brain injury [40, 41]. The content of full-length neurocan (expressed relative to a truncated form of this protein) also was not significantly different in olomoucine-treated rats compared with the vehicle-treated rats (Fig. 4C; Additional file 1: Fig. S3).

**Fig. 5:**
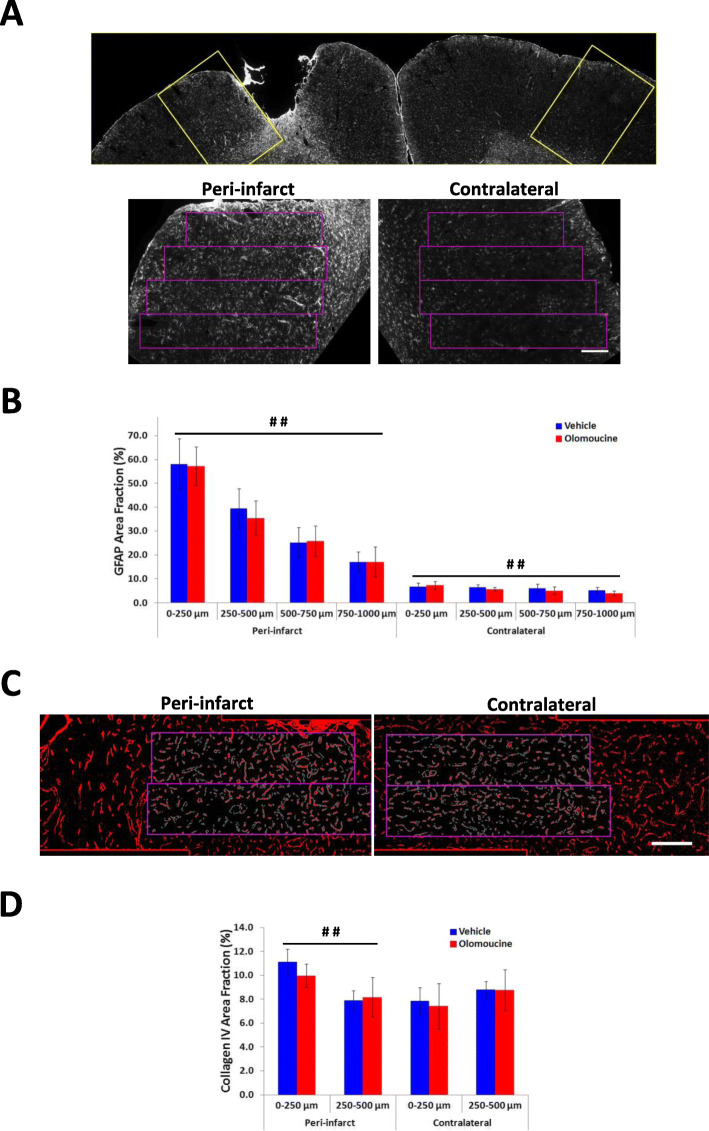
Immunolabelling for GFAP and collagen IV at 29 days after stroke*.* **A** The low power image in the upper panel shows the typical pattern of GFAP labelling, with increases extending more than 1 mm into the grey matter surrounding the infarct. Prominent increases were seen on the rim of the infarct, in grey matter at the base of the infarct and in underlying white matter. The rectangles in this image identify the regions shown in the lower images. The rectangles in the lower images show the tissue that was analyzed. The narrow band of increased immunolabelling above the rectangles corresponds to the developing glial scar. **B** Effect of olomoucine treatment on the area fraction of GFAP immunolabelling. Olomoucine treatment did not significantly affect the area fraction of GFAP labelling in either hemisphere. There was a marked effect of distance from the lesion on immunolabelling in peri-infarct tissue (*p* < 0.01) and also a small but significant effect of sampling location in the contralateral cortex (*p* < 0.01). **C** Collagen IV immunolabelling. The rectangles delineate the regions in which area fraction of immunolabelling was assessed. **D** Effect of olomoucine treatment on the area fraction of collagen IV immunolabelling. There was a significant effect of distance from the lesion on immunolabelling in peri-infarct tissue (*p* < 0.01) but no significant differences in equivalent contralateral tissue. The area fraction of immunolabelling was not significantly affected by olomoucine treatment in either hemisphere. The scale bars in **A** and **C** = 200 μm. Results in **B** and **D** are shown as mean ± SD (*n* = 6 per group) and were analyzed using two-way analysis of variance. ##*p* < 0.01 for the effect of distance from the infarct or of location in the contralateral cortex

The brains removed from rats at 29 days after completion of the assessment of forepaw function were analyzed for possible changes in immunolabelling for GFAP and collagen IV. Increases in GFAP expression are still prominent at this time in the developing glial scar located at the edge of the infarct and also remain elevated in the tissue exhibiting essentially normal neuronal viability that extends at least one millimetre into cortical grey matter from the rim of the scar (Fig. 5A). The changes in GFAP expression in peri-infarct tissue beyond the developing scar are indicative of an ongoing reactivity in astrocytes that can more directly influence adaptive responses in neighbouring neurons [18]. We investigated possible effects of the olomoucine treatment on this response by measuring the area fraction of GFAP immunolabelling in successive 250-μm-wide regions extending 1 mm from the infarct (Fig. 5A, B), an approach that has previously detected effects of other treatments on this cell population [37, 49]. As expected, GFAP immunolabelling was much higher in this tissue compared with equivalent tissue from the contralateral hemisphere. There was no effect of olomoucine treatment on the GFAP response in either hemisphere. GFAP labelling was significantly affected by distance from the lesion in peri-infarct tissue and location in the contralateral cortex. Post-hoc analysis (Tukey HSD test) revealed significant differences in pair-wise comparisons between all regions in peri-infarct tissue except for that at 500 to 750 μm compared with 750 to 1000 μm. In the contralateral hemisphere, a significant difference was seen between tissue at 0 to 250 μm compared with 750 to 1000 μm.

Collagen IV is expressed by endothelial cells in capillaries and has been shown to increase in peri-infarct tissue in association with angiogenesis, which can contribute to adaptive responses [8, 50]. The area fraction of collagen IV immunoreactivity was higher in peri-infarct tissue compared with the contralateral hemisphere (Fig. 5C, D). Again, there was no significant difference between treatment groups in either hemisphere.

## Discussion

The main finding from this study is that olomoucine treatment initiated early after induction of stroke by photothrombosis modified functional recovery. Interestingly, the effects of the treatment on recovery were markedly task dependent, with a worse outcome on performance in a forelimb placing test but improved recovery in both total success and first-trial success in a skilled reaching test. The altered patterns of recovery following olomoucine treatment occurred in the absence of a significant change in infarct size. Thus, the effects of olomoucine were apparently not due to changes in gross tissue damage and probably arose from modifications in the downstream cellular responses to this damage. Consistent with this possibility, olomoucine treatment significantly reduced at least one major component of reactive astrogliosis, as evidenced by reductions in tissue vimentin content in peri-infarct tissue at 1 week after the stroke. In contrast, the treatment had little effect on key features of the responses of microglia and macrophages in peri-infarct tissue.

The two tests used to assess forelimb function in the present study differ in several ways, including the complexity of the task and a requirement for training, that could contribute to the differential influence of olomoucine treatment on recovery. The forepaw placing test, which recovered more slowly following olomoucine treatment, requires a motor response to stimulation of the vibrissae. The effects of interventions on recovery of this task are often similar to that of other general measures of limb motor function or broader assessments of neurological function (for examples see: [51–53]). In line with our previous findings [11], this test was particularly sensitive to the lesion induced by photothrombosis. Induction of stroke produced near complete loss of this response in all rats. Rats exhibited substantial recovery over the next 4 weeks, although most did not regain baseline performance at 28 days. No specific training was required to elicit this response and progressive recovery occurred without additional training beyond the weekly testing sessions.

The skilled reaching task, which showed a better recovery following olomoucine treatment, is a complex learned response that requires targeted movements and fine motor control in the forelimb. Rats in the present study were tested 12 times between 6 and 29 days. The multiple sessions in this period probably contributed to the extent of recovery as post-stroke training has been found to promote spontaneous recovery [6] and improve the response to other interventions [44, 54, 55].

Previous investigations of the effects of several other interventions on functional recovery in rodent models of stroke have also identified marked differences in the effects on general assessments of motor function or gross neurological performance compared with skilled tasks. However, the responses to different tests of limb function are generally not as markedly divergent as that following olomoucine treatment. A pattern broadly consistent with the effects of olomoucine was seen in response to a treatment involving forced use of the affected forelimb following both ischemic and hemorrhagic stroke [51, 55, 56]. Forced forelimb use improves recovery on skilled reaching tasks but does not usually improve more general measures of forelimb function or general motor performance. Indeed, an early imposition of forced use of the affected forelimb in some studies impaired the recovery of measures of general neurological function, including a forelimb placing test [57, 58]. In contrast, environmental enrichment following stroke commonly promotes recovery in general assessments of forelimb function but this intervention alone is typically ineffective in improving outcomes in skilled reaching tests and similar tasks [52, 59–61].

Multiple changes in the brain are thought to contribute to recovery following stroke. This includes altered neuronal activity, axonal sprouting, synaptogenesis, neurogenesis and gliogenesis within the peri-infarct tissue as well as modifications to the activity and connectivity of neurons at more distant locations [5–8]. The specific elements of these responses underlying differential outcomes between skilled tasks and general motor function following some treatments have not been well defined. A recent investigation found that improvements in skilled reaching induced by forced limb use together with skilled forelimb training after induction of photothrombotic stroke were associated with increased connectivity of preserved corticospinal neurons in remaining motor cortex adjacent to the infarct [55]. Thus, changes affecting the environment or activity of these cells in peri-infarct tissue could potentially modify recovery of skilled tasks by altering axonal sprouting.

Photothrombosis occludes vessels in the pia and cortical parenchyma, producing a region of permanent ischemia [31]. Under the conditions used in the present study, this approach generates a maximal infarct volume within 24 h that is essentially maintained at 3 days and then slowly contracts [11]. The lack of a significant effect of olomoucine on infarct volume at 3 days strongly supports the conclusion that the differences in recovery were not due to alterations in the severity of the initial tissue damage but rather arose from modifications of the downstream cellular responses. This situation contrasts markedly with previous reports in which the cell cycle inhibitor, roscovitine, improved recovery in general tests of motor function, a change that was associated with decreases in infarct volume [23, 25, 26]. The use of a model involving temporary middle cerebral artery occlusion and earlier initiation of treatment in these previous studies are likely to have contributed to the different outcomes compared with our investigation.

Our use of photothrombotic stroke rather than a model involving temporary arterial occlusion also has implications for the potential relevance to stroke in humans. Early reperfusion results in major differences in the progression to tissue damage when evaluated in animal models [2] and is also likely to alter downstream cellular responses. The increased clinical use of thrombolysis and clot retrieval to treat acute stroke over the last decade has increased the number of cases in which blood flow is returned to the ischemic tissue within the first few hours following onset [62]. However, many patients do not receive these interventions. Spontaneous reperfusion can develop without such treatments but this usually does not occur for many hours to days [63]. The permanent ischemia induced by photothrombotic stroke provides a better model than short-term middle cerebral artery occlusion for those patients in which local disruption to blood flow is permanent or restoration of flow is markedly delayed.

Proliferation of microglia is an important component of the response of these cells in peri-infarct tissue in the first few days after stroke [12, 19, 23]. Thus, these cells were considered a likely target for the olomoucine treatment that was given at 1 h and 24 h after the photothrombosis. Features of Iba1 immunolabelling were used as the primary measure of microglial reactivity as this allowed us to reliably limit the analysis to cells in the peri-infarct tissue. Tissue of interest around the infarct was reproducibly defined on the basis of the presence of near-normal NeuN immunolabelling in the same sections. Iba1 immunohistochemistry has been widely used to detect microglial reactivity in peri-infarct tissue following stroke but most studies have relied on qualitative comparisons of the extent or intensity of immunolabelling. In only a few investigations have features of the responses of the Iba1-positive cells been quantified [10–12].

In the present study, both the circularity of Iba1-immunopositive cells and the area fraction of Iba1-immunolabelling were analyzed at 3 days when these responses are at or near the maximum. Circularity increases primarily as a result of morphological changes in the cell associated with activation of the microglia. It reaches maximal values by 24 h in peri-infarct tissue and these increases are largely maintained at 3 days [11]. The area fraction of Iba1 immunoreactivity in peri-infarct tissue is markedly increased by 3 days, primarily reflecting an increase in cell numbers [11]. The Iba1 area fraction remains similarly increased at 7 days. The local proliferation of the microglia is an important contributor to this increase [12, 19, 23], with migration of microglia from surrounding tissue probably also involved. The movement of microglia towards sites of tissue damage is a well characterized response of these cells following activation [64]. This migration is commonly proposed to contribute to increases in the numbers of these cells in the infarct and peri-infarct tissue [13, 14]. However, to our knowledge, the relative contribution of proliferation and migration to these changes following stroke has not been determined.

Olomoucine treatment produced a small increase in the circularity of Iba1-immunolabelled cells and a small decrease in the Iba1 area fraction in peri-infarct tissue. However, similar changes also developed in tissue from the contralateral hemisphere. Thus, the treatment with olomoucine apparently did not lead to specific modifications of these key responses of peri-infarct Iba1-positive cells to the ischemia-induced tissue damage.

Tissue macrophages derived from circulating monocytes express Iba1 and contribute to the changes in Iba1-positive cells in peri-infarct tissue. However, these macrophages are apparently present at much lower numbers than microglia in the peri-infarct tissue at 3 days after stroke. A recent detailed analysis detected tissue macrophages derived from circulating monocytes in the infarct and peri-infarct tissue at 3 days after photothrombotic stroke in mice [65]. These cells accounted for approximately 30% of Iba1-positive cells in tissue immediately surrounding the infarct but the sampling included tissue that was excluded from our analysis because of neuronal loss. Macrophages contributed less than 15% to the total Iba1-positive cells in neighbouring tissue that corresponds to much of the peri-infarct tissue analyzed in our investigations. Other studies have reported that macrophages accounted for less than 10% of the Iba1-positive cells in peri-infarct tissue following photothrombotic stroke [66] and permanent middle cerebral artery occlusion [67]. Thus, microglia have been consistently identified as the main contributors to the Iba1 immunoreactivity that we evaluated in peri-infarct tissue at 3 days. Alterations induced in the microglia by the olomoucine treatment are unlikely to have been masked by changes in the responses of blood-derived macrophages.

Analysis of the numbers of CD68-positive cells indicated that olomoucine also had limited effects on an additional aspect of the response of microglia surrounding the infarct. CD68 is a member of the LAMP family of proteins that is predominantly localized intracellularly in the membranes of lysosomes and late endosomes [45, 68, 69]. Within the first few days after stroke induction, CD68-positive cells are found in outer parts of the infarct, with smaller numbers in the peri-infarct tissue immediately adjacent to the lesion (Fig. 3C [11, 70];). At 3 days after photothrombotic stroke in rats, most CD68-positive cells are microglia [70]. Blood-derived macrophages become more involved by day 6. The presence of CD68 has been associated with phagocytosis in macrophages [68, 69] but the role of CD68-positive cells in the peri-infarct tissue has not been determined. Decreases in the numbers of peri-infarct CD68-positive cells have been seen following a diverse range of interventions that have improved functional recovery following stroke [11, 43–45], raising the possibility that these cells might limit positive adaptive responses in peri-infarct neurons or their connections. No such changes were detected in peri-infarct tissue following olomoucine treatment. A doubling in the small numbers of cells expressing CD68 was seen in cortical tissue contralateral to the lesion, again suggesting that olomoucine had subtle effects on the properties of microglia in the brain without specifically affecting the responses of peri-infarct cells.

The numbers of cells immunopositive for the cell cycle protein, Ki67, was also not significantly affected by olomoucine treatment when assessed at 3 days after stroke. As Ki67 expression develops early in the cell cycle [71], a block of proliferation induced by olomoucine was not expected to directly influence Ki67-positive cells. However, changes could arise when assessed at 3 days due to downstream modifications of the proliferation of cells not directly affected by the treatment, as was found between 1 and 7 days following treatment with the cell cycle inhibitor, roscovitine [23]. The proportion of these cells that were also immunopositive for Iba1 in our study (38 ± 8% within the first 250 μm from the infarct when averaged over all rats) is similar to numbers of Iba1-positive proliferating cells detected using bromodeoxyuridine labelling at 3 to 4 days after photothrombotic stroke in rats [12] or temporary middle cerebral artery occlusion in mice [19]. This subpopulation of proliferating cells was not significantly affected by the olomoucine treatment.

Together, the measures of Iba1, CD68 and Ki67 immunoreactivity strongly suggest that treatment with olomoucine did not substantially modify key elements of the microglial response in peri-infarct tissue including the marked morphological changes, proliferation, migration towards the tissue damage and CD68 expression.

The most prominent change seen in glial-cell related parameters in response to olomoucine treatment was in vimentin, a cytoskeletal protein that is markedly increased during reactive astrogliosis. Vimentin content was reduced by 30% in samples of tissue that included the infarct and neighbouring peri-infarct tissue at 7 days after stroke induction, when increases in expression of this protein are near maximal [11, 46, 47]. Olomoucine treatment did not significantly affect the expression of another cytoskeletal protein, GFAP, or full-length neurocan, suggesting that all components of reactive astrogliosis were not equally affected. However, the different outcome for the two cytoskeletal proteins might reflect a greater sensitivity of vimentin compared with GFAP content in detecting the treatment-induced changes following photothrombotic stroke. Vimentin expression in normal mature brain is low and is essentially restricted to endothelia and perhaps other cells surrounding blood vessels [72, 73]. The expression increases in astrocytes immediately surrounding the infarct within the first 2 days. By 7 days, vimentin content is many times greater than that seen in equivalent tissue in the contralateral cortex or in the cortex prior to stroke (Fig. 4A, [11, 46, 47]). Changes in GFAP expression largely parallel those of vimentin. However, this protein is also expressed by astrocytes in the absence of tissue damage [46, 48, 49]. Thus, the proportional increases in response to stroke are substantially smaller. This difference in sensitivity between the two markers is potentially more of an issue with photothrombotic stroke that typically produces a smaller infarct and more limited cellular responses than are seen in models involving middle cerebral artery occlusion.

A reduction in aspects of reactive astrogliosis, indicated by the decreased vimentin, is broadly consistent with a previous report in which GFAP content was reduced in the absence of significant changes in infarct volume when olomoucine treatment was initiated early after induction of middle cerebral artery occlusion [28]. Modifications to astroglial reactivity could arise either from direct effects of olomoucine on astrocytes or as a result of modifications of signals from microglia or the damaged tissue that help initiate astrocytic reactivity. The very limited effects of olomoucine on the measured microglial properties suggest that altered signalling from these cells is less likely to be involved. Astrocytic proliferation develops within a few days of stroke and is almost completely restricted to a subpopulation of cells within 200 μm of the infarct [12, 19, 22]. Although the proliferating cells are only a minority of astrocytes at this location, studies of other forms of central nervous system injury [15, 21] and indirect evidence in stroke [22, 74] indicate that these cells are critical for normal development of the astroglial scar [18]. Alterations in scar formation could account for the reductions in vimentin content seen in the present study although secondary effects of the drug treatment on non-proliferating astrocytes outside of the scar cannot be ruled out.

Interventions that decrease aspects of reactive astrogliosis can impair or enhance functional recovery following stroke [18], reflecting the multiple roles of these cells and the complexity of their responses. Nonetheless, reductions in astrocytic responses more commonly lead to decreased recovery, at least when assessed using measures of general neurological function [75–80]. Thus, alterations in reactive astrogliosis, as indicated by decreased vimentin content, could have contributed to the decrease in recovery on the forelimb placing response in the present study. Most studies of treatments that reduce elements of reactive astrogliosis have not examined possible effects on skilled reaching tasks. In one notable exception, double-knockout mice lacking expression of GFAP and vimentin exhibited impaired recovery in a test of grid walking and a skilled reaching test following photothrombosis in the absence of effects on infarct volume [79]. The lack of both of these cytoskeletal proteins results in much more extensive changes in astroglial reactivity than those induced by olomoucine, a difference that could contribute to the different effects on recovery of the skilled reaching task.

The treatment protocol in which olomoucine was injected twice in the initial 24 h after stroke was chosen to increase chances of influencing the early proliferation in peri-infarct glia and also because this protocol had been shown previously to modify a component of reactive astrogliosis without altering infarct volume [28]. Nonetheless, alterations in the proliferation of cells at other sites in the brain [81, 82] or of cells involved in the peripheral immune response to stroke [83, 84] might also have contributed to the observed effects of olomoucine on recovery. Neural precursor cells in the subventricular zone can contribute to recovery in tests of general motor function following stroke, [81, 82, 85]. Proliferation of these precursor cells in the subventricular zone begins by day 2 but increases greatly over the next 2 weeks [81, 86, 87]. The delayed development of most of this proliferation suggests limited opportunities for modification by the early olomoucine treatment. Furthermore, although small numbers of new neurons are produced in peri-infarct tissue, the integration of these cells and involvement in recovery develops over many weeks [82, 85]. Thus, disruption of these responses seems unlikely to account for the decreased recovery of the paw placement test that was detectible at 14 days after stroke in our study. Neural precursor cells also generate small numbers of astrocytes in peri-infarct tissue within the first few days after stroke [88]. Effects of olomoucine on the production of this subpopulation of astrocytes might have contributed to the effects of olomoucine on reactive astrogliosis and recovery.

## Conclusions

Olomoucine treatment early after photothrombotic stroke modified recovery of motor function in a task-dependent manner without affecting infarct volume. To our knowledge, this is the first demonstration that treatment with a cell cycle inhibitor can influence post-stroke recovery in the absence of significant effects on size of the infarct. The findings add to evidence for differences in the cellular responses underlying recovery of general neurological function compared with skilled tasks requiring training. The study further demonstrated that olomoucine treatment leads to reductions in an important component of reactive astrogliosis in peri-infarct tissue. In contrast, there was little effect on key properties of peri-infarct microglia even though some of these cells divide early after infarct development. Alterations induced in aspects of reactive astrogliosis could modify adaptive responses of neurons in peri-infarct tissue and influence recovery of neurological function.

## Supplementary Information

**Additional file 1: Fig. S1.** Western blot of GFAP in samples of the infarct plus peri-infarct tissue at 7 days after photothrombotic stroke. Rats were treated with olomoucine (O) or vehicle (V). The investigator responsible for the Western blotting was blinded as to the treatment of the rats and had no control over the order of the samples. The numbers show the total protein for each sample as determined from chemiluminescence after transfer to the polyvinylidene fluoride membrane expressed relative to the mean intensity of all the lanes. Samples from olomoucine-treated rats in lanes 5 and 10 show evidence of marked degradation of the GFAP compared with the other samples and were not used in the subsequent analysis. **Fig. S2.** Unprocessed images of Iba1 immunolabelling in coronal sections of the peri-infarct tissue and equivalent tissue from the contralateral cortex from a rat treated with olomoucine and one treated with vehicle. The upper edges of the images from the peri-infarct tissue run parallel to the edge of the infarct as shown in Fig. 3A. Tissue excluded from analysis based on the loss of NeuN immunoreactivity extends approximately 150 μm into the images. The scale bar represents 500 μm. **Fig. S3.** Western blots of vimentin and neurocan in samples of the infarct plus peri-infarct tissue at 7 days after photothrombotic stroke. Rats were treated with olomoucine (O) or vehicle (V). The investigator responsible for the Western blotting was blinded as to the treatment of the rats and had no control over the order of the samples. The numbers in the vimentin blot show the total protein for each sample as determined from chemiluminescence after transfer to the polyvinylidene fluoride membrane expressed relative to the mean intensity for all the lanes. In the neurocan blot, the full-length protein (approximately 250 kDa) and major fragment (approximately 150 kDa) detected by the antibody are indicated. Results for this protein were determined as a ratio of the full length protein to the fragment. The break in the blots is due to the removal of lanes that were not used in the analysis of vimentin or neurocan content.

## Data Availability

The datasets used and/or analyzed during the current study are available from the corresponding author on reasonable request.

## Abbreviations

PBS: Phosphate-buffered saline
ROIs: Regions of interest
