# Supplementary material for: Differential effects of the cell cycle inhibitor, olomoucine, on functional recovery and on responses of peri-infarct microglia and astrocytes following photothrombotic stroke in rats
Source: J Neuroinflammation. 2021 Jul 31;18:168. doi: 10.1186/s12974-021-02208-w (PMC8325288; doi:10.1186/s12974-021-02208-w)
Supplement: Supplementary file 1 — Additional file 1: Fig. S1. Western blot of GFAP in samples of the infarct plus peri-infarct tissue at 7 days after photothrombotic stroke. Rats were treated with olomoucine (O) or vehicle (V). The investigator responsible for the Western blotting was blinded as to the treatment of the rats and had no control over the order of the samples. The numbers show the total protein for each sample as determined from chemiluminescence after transfer to the polyvinylidene fluoride membrane expressed relative to the mean intensity of all the lanes. Samples from olomoucine-treated rats in lanes 5 and 10 show evidence of marked degradation of the GFAP compared with the other samples and were not used in the subsequent analysis. Fig. S2. Unprocessed images of Iba1 immunolabelling in coronal sections of the peri-infarct tissue and equivalent tissue from the contralateral cortex from a rat treated with olomoucine and one treated with vehicle. The upper edges of the images from the peri-infarct tissue run parallel to the edge of the infarct as shown in Fig. 3A. Tissue excluded from analysis based on the loss of NeuN immunoreactivity extends approximately 150 μm into the images. The scale bar represents 500 μm. Fig. S3. Western blots of vimentin and neurocan in samples of the infarct plus peri-infarct tissue at 7 days after photothrombotic stroke. Rats were treated with olomoucine (O) or vehicle (V). The investigator responsible for the Western blotting was blinded as to the treatment of the rats and had no control over the order of the samples. The numbers in the vimentin blot show the total protein for each sample as determined from chemiluminescence after transfer to the polyvinylidene fluoride membrane expressed relative to the mean intensity for all the lanes. In the neurocan blot, the full-length protein (approximately 250 kDa) and major fragment (approximately 150 kDa) detected by the antibody are indicated. Results for this protein were determined as a ratio of the full lengt [file 12974_2021_2208_MOESM1_ESM.docx]

Figure S1

**GFAP**

**V - Vehicle treated O - Olomoucine treated**


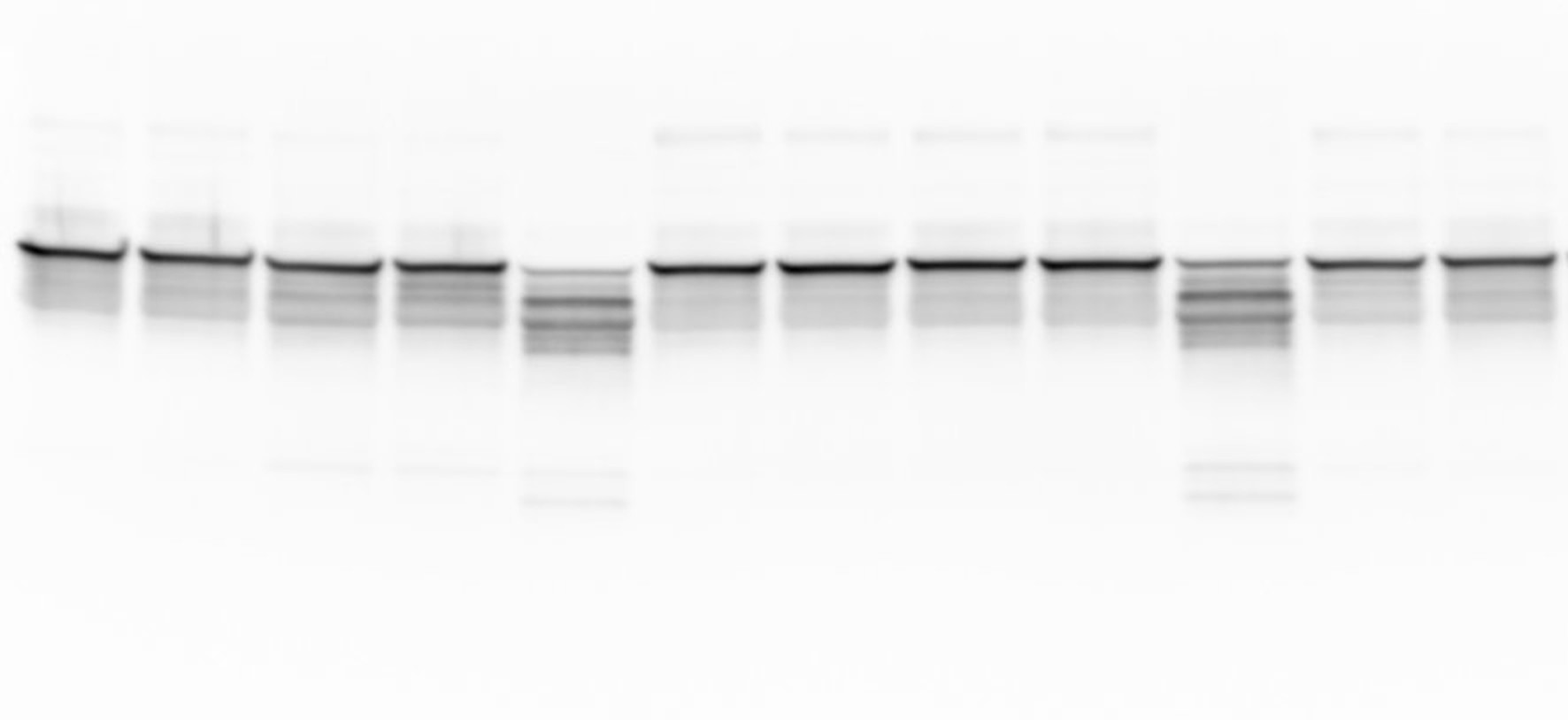


**GFAP**

**Total Protein**

**1.00 0.88 1.21 0.99 1.09 0.96 1.05 0.95 0.89 1.00 1.07 0.90**

**O O O V O O V V V O V V**

Figure S2

**Contralateral**

**Peri-infarct**

**Iba1**


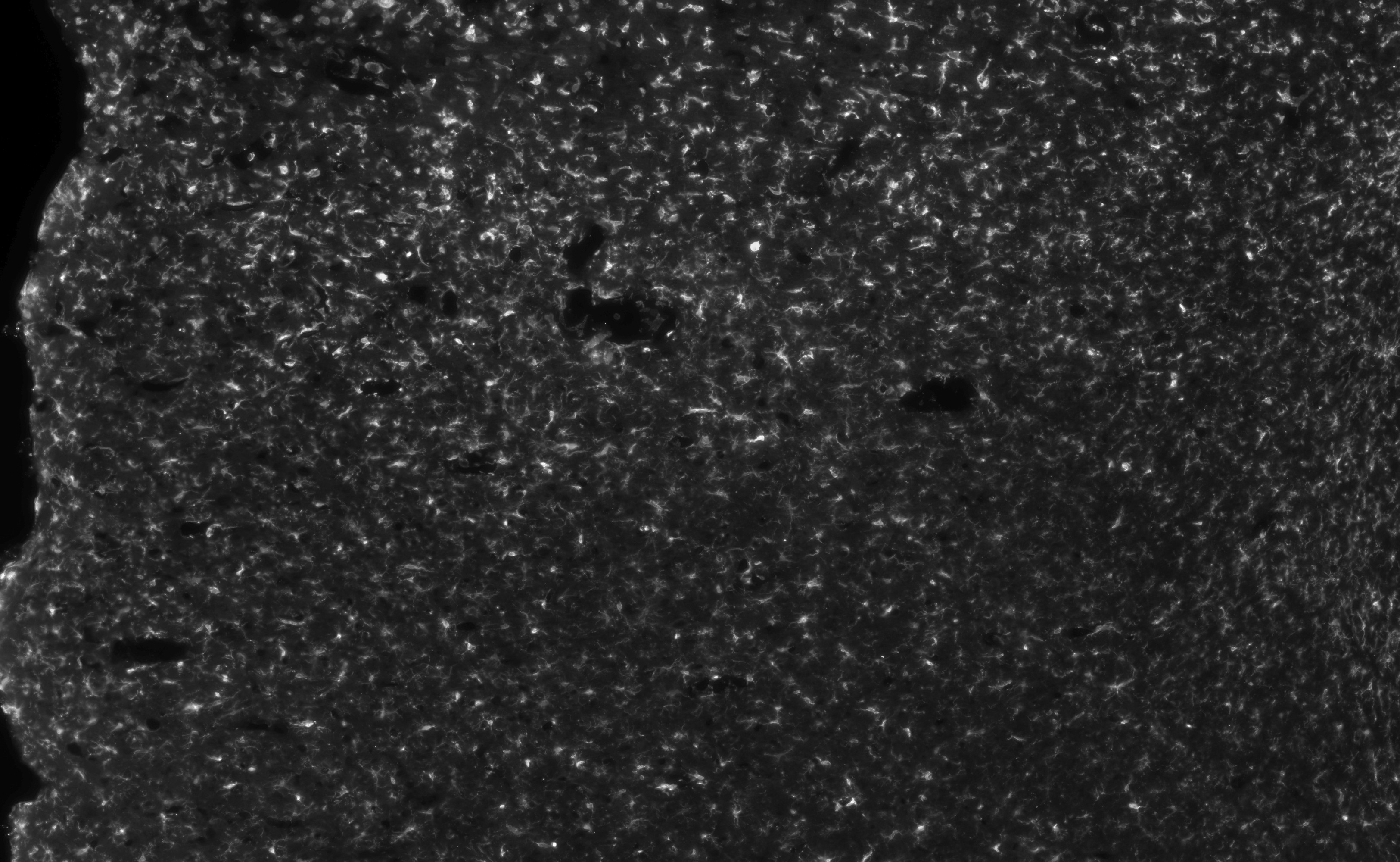

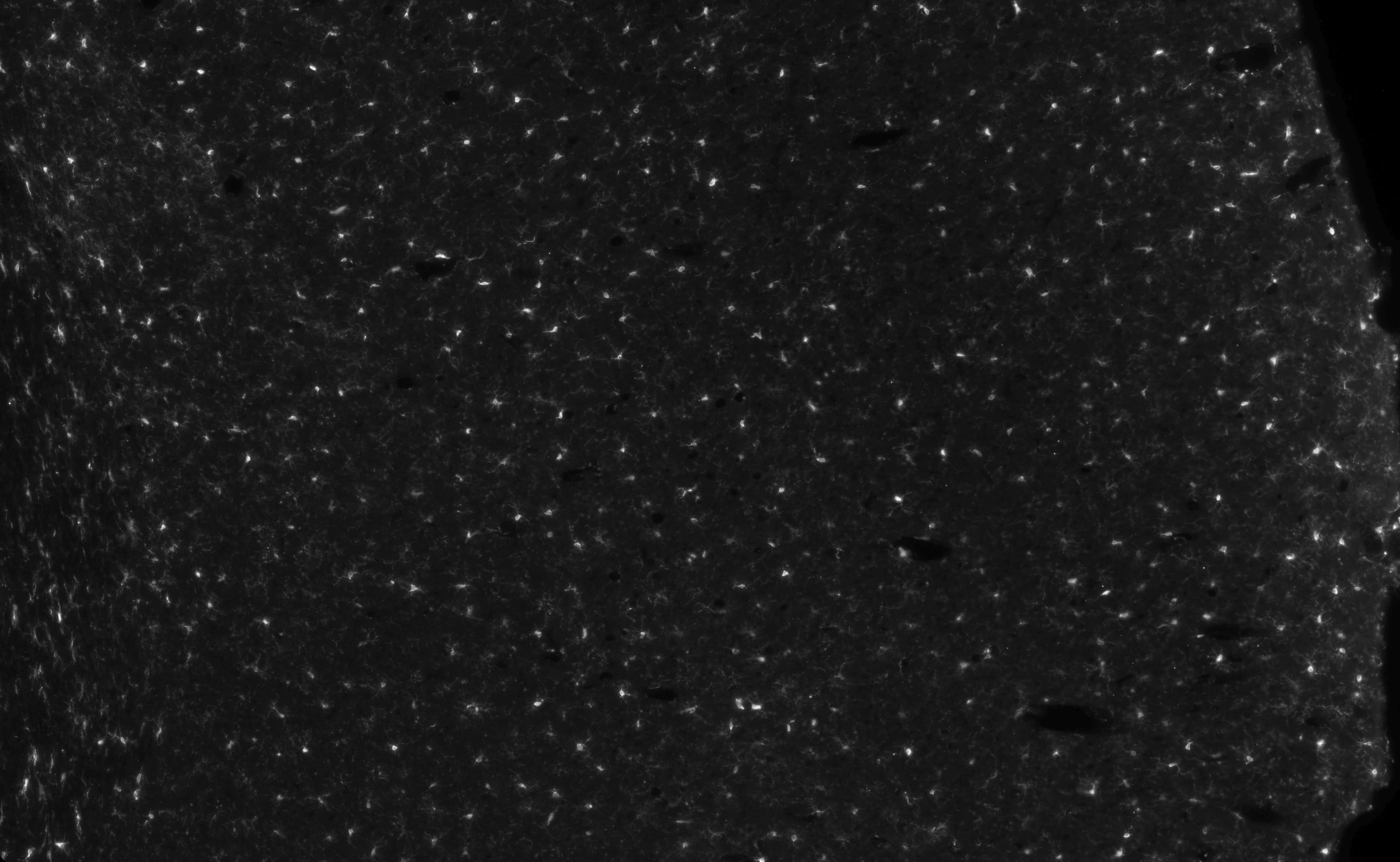


**Vehicle**


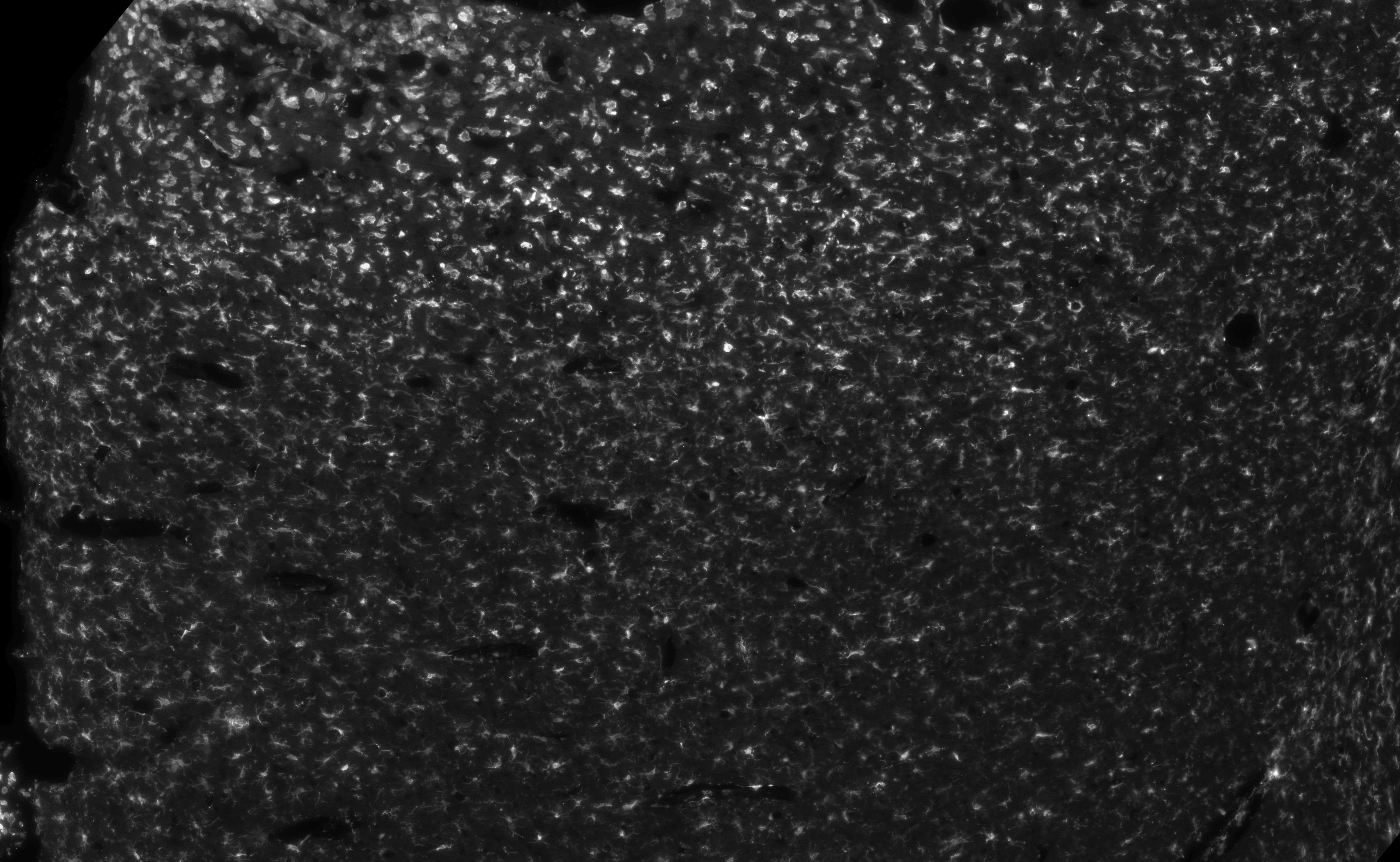

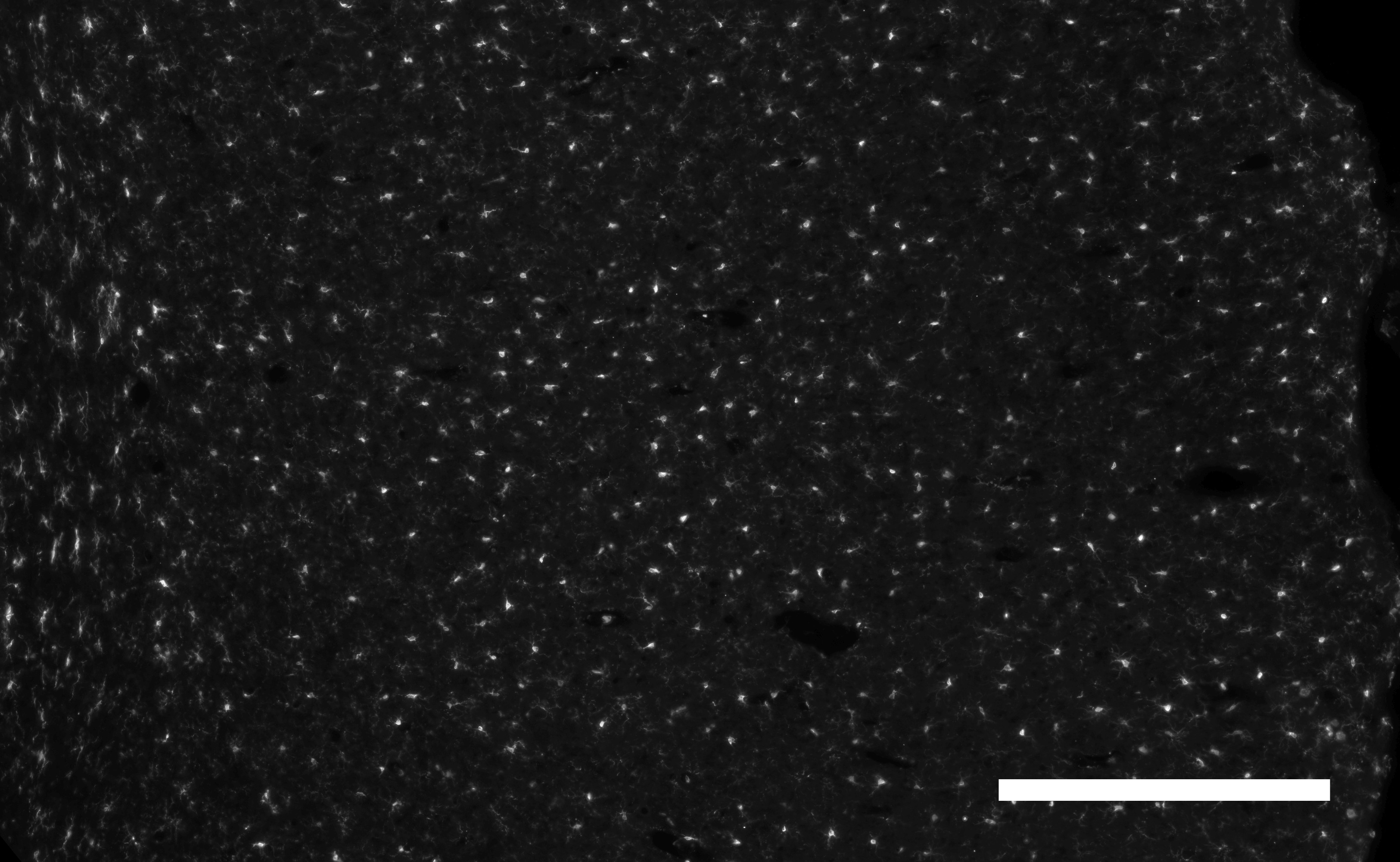


**Olomoucine**

Figure S3

**Vimentin**

**A**

**V - Vehicle treated O - Olomoucine treated**


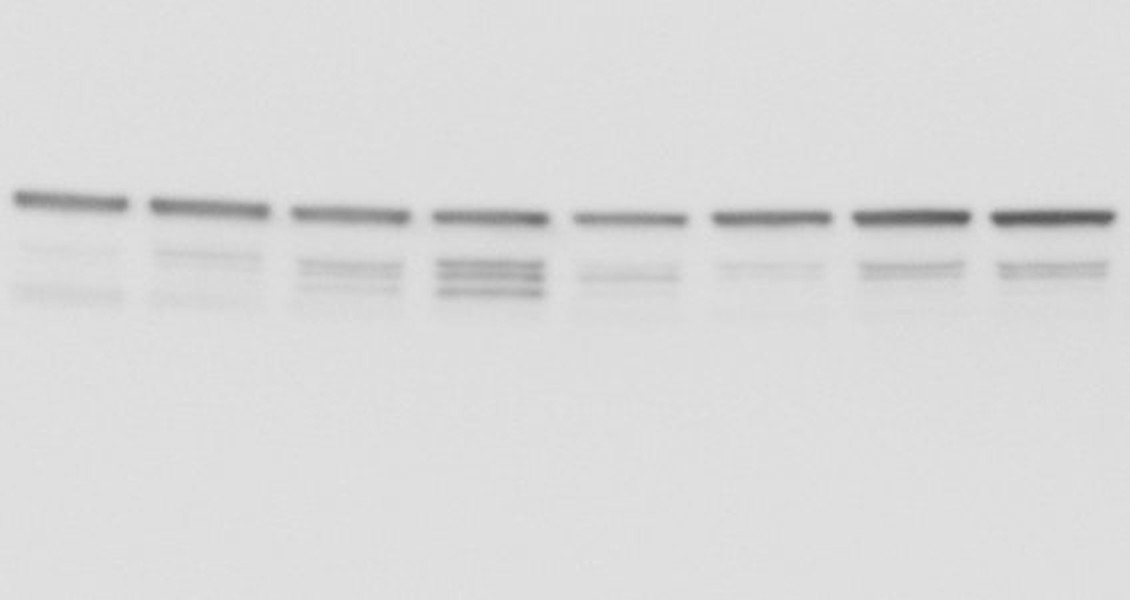

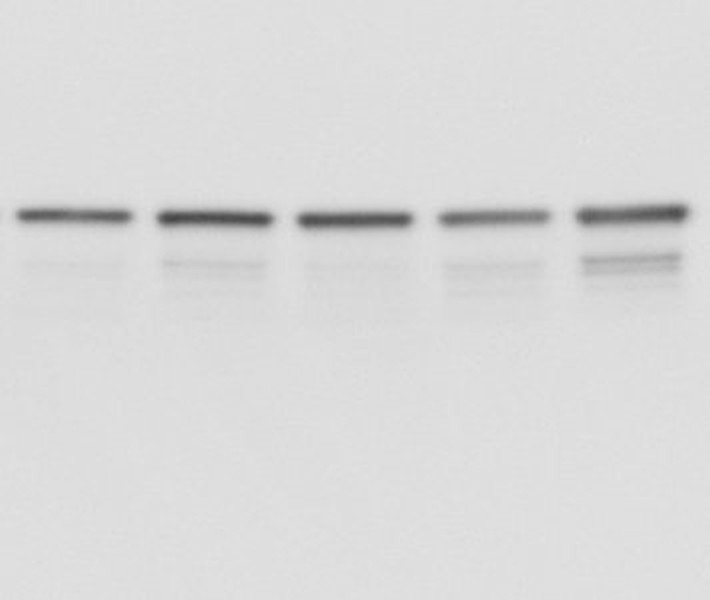


**Total Protein**

**Vimentin**

**1.01 0.91 1.32 1.01 1.01 1.03 1.18 1.31 0.91 0.81 0.87 0.92 0.72**

**O O O V O O V O V V V V V**

**B**

**Neurocan**

**V - Vehicle treated O - Olomoucine treated**


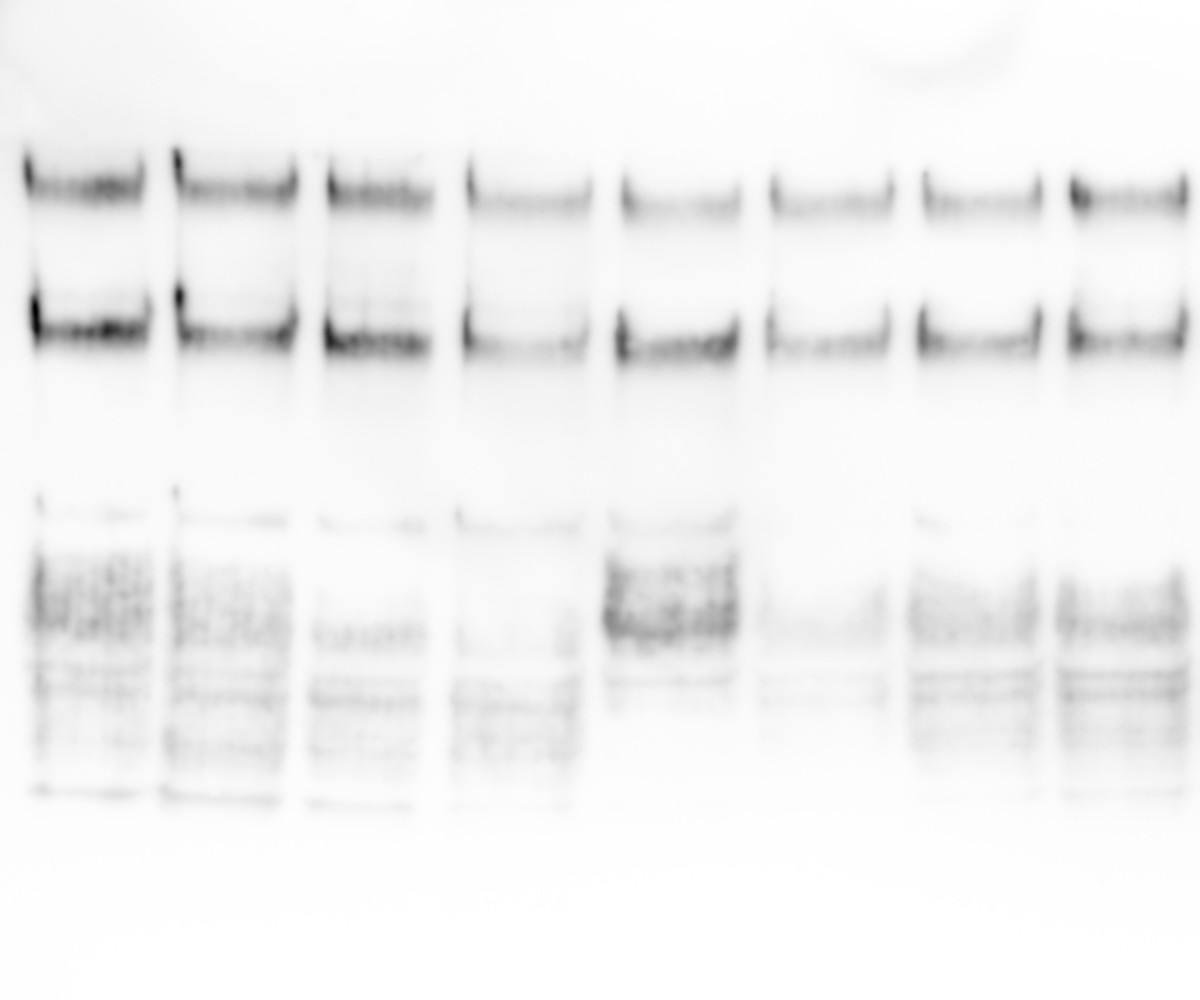

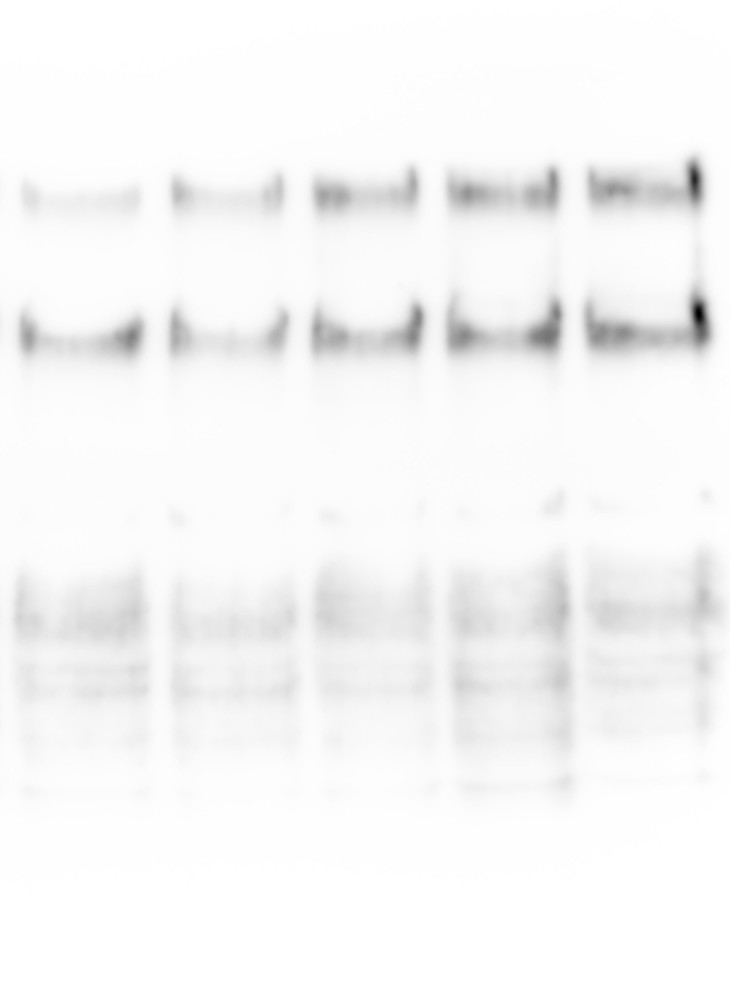


**250 kDa**

**150 kDa**

**O O O V O O V O V V V V V**
